# A Pilot Randomized Trial of Intradialysis Yoga for Patients With End-Stage Kidney Disease

**DOI:** 10.1016/j.ekir.2022.11.007

**Published:** 2022-11-19

**Authors:** Ann Herron, Kerri L. Cavanaugh, Ebele M. Umeukeje, T. Alp Ikizler, Gurjeet Birdee

**Affiliations:** 1 Division of Nephrology and Hypertension, Vanderbilt University Medical Center, Nashville, Tennessee, USA; 2 Department of Physical Medicine and Rehabilitation, Vanderbilt University Medical Center, Nashville, Tennessee, USA

**Keywords:** hemodialysis, mind-body, yoga

## Introduction

Exercise has been shown to be beneficial for patients receiving maintenance hemodialysis (MHD), improving quality of life and self-reported physical functioning.^1^ However, application of exercise programs outside of clinical studies is difficult given variable adherence and low exercise counseling among nephrologists.^2^ Mind-body practices such as yoga, tai chi, deep breathing, and meditation represent an alternative to conventional exercise programs. Very few studies have evaluated the clinical efficacy of mind-body practices during MHD. Yoga is a mind-body practice that uses various postures, meditations, and breathing exercises to promote physical, mental, and spiritual well-being. The safety an intradialytic yoga (IDY) program has previously been shown.^3^ However, to our knowledge, no studies have been done evaluating the effects of yoga performed during MHD. This study aims to evaluate the feasibility of conducting a randomized clinical pilot study to measure the effects of an IDY on quality of life, self-efficacy, and physical performance among MHD patients. Participants were randomized to IDY or a comparison group that received an educational course called Kidney School. Methods including description of the yoga intervention and participant characteristics are detailed in the Supplementary Methods (Supplementary Tables S1 and S2).

## Results

**Figure 1:**
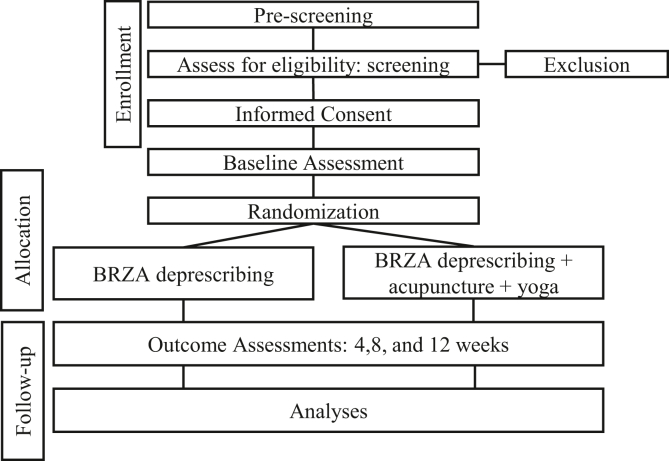
CONsolidated Standards of Reporting Trial flow diagram.

Patients were enrolled in the study from July 2015 to September 2016, with a total of 363 patients assessed for eligibility (Figure 1). Of these, 294 patients were excluded, and a total of 69 patients were randomized to either the IDY group or the education group. One patient was lost to follow-up in each arm. Questionnaires were completed by 51 participants at 6 weeks and by 53 participants at 12 weeks, and the 6-minute walk test was completed by 48 participants at 12 weeks. Using intention-to-treat analysis, 34 yoga patients and 33 education patients were analyzed.

Participant demographics are described in Supplementary Methods (Supplementary Table S2). The median age (interquartile range) was 58 (50, 64) years, approximately half the patients were female, and a large majority (88%) of the participants were African American.

Patients in the IDY group attended an average of 32 out of 35 MHD treatments, representing more than 90% participation in offered sessions. During the study, 5 adverse events occurred requiring yoga to be discontinued during MHD. These episodes included light-headedness (2 patients), vascular access dysfunction (1 patient), hypotension (1 patient), and cramping (1 patient). On review, these events were not attributed to the act of practicing yoga. No adverse events were attributed to the educational program.

The results for the primary (Kidney Disease Quality of Life Physical Function and Kidney Disease Quality of Life Mental Function) and secondary outcomes (physical performance and Personal-Disclosure Mutual-Sharing self-efficacy) are presented in Table 1. There were no significant changes in physical function by patient-reported (kidney disease quality of life) or performance measures (6-minute walk tests) for either treatment arms. From baseline to 6-weeks, mental health had a statistically nonsignificant improvement among IDY group and decreased in the education group. At 12-weeks, mental health continued to be numerically higher in the IDY group than in the education group. In an adjusted analysis considering baseline values, gender, and diabetes, the IDY group had nonsignificant improvements in mental health versus the education group (−9.21, CI −20.29 to 1.86]. No differences were observed over the study period regarding self-efficacy.

**Table 1:** Adjusted estimated treatment effects for primary and secondary outcomes

Outcome by treatment	Baseline^a^	6 wks^a^	12 wks^a^	Estimated treatment effect (95% CI)^b^
KDQOL Physical Function score				
Yoga	40.0 (29.1, 51.1)	40.5 (30.1, 51.1)	39.0 (30.2, 49.4)	
Education	37.2 (29.8, 44.9)	34.8 (25.6, 41.5)	39.1 (30.2, 46.1)	
				−0.53 (−12.00, 10.93)
KDQOL Mental Health score				
Yoga	51.6 (46.3, 59.3)	55.2 (51.3, 59.7)	53.5 (45.9, 58.8)	
Education	53.5 (41.9, 59.0)	43.6 (36.1, 57.1)	50.2 (43.5, 58.4)	
				−9.21 (−20.29, 1.86)
6 min walk test (m)				
Yoga	313 (226, 369)		302 (193, 375)	
Education	312 (228, 346)		300 (261, 323)	
				49.55 (−31.31, 130.40)
PDSM self-efficacy score				
Yoga	3.25 (3.00, 3.50)		3.12 (3.00, 3.44)	
Education	3.00 (3.00, 3.16)		3.00 (3.00, 3.25)	
				−0.04 (−0.24, 0.16)

CI, confidence interval; KDQOL, Kidney Disease Quality of Life; PDSM, Personal-Disclosure Mutual-Sharing.

a Median [Interquartile range].

b Adjusted for baseline values, gender, and diabetes status to account for imbalances in randomization.

## Discussion

This randomized pilot and feasibility study demonstrated that a yoga intervention can be implemented during MHD while conducting a randomized clinical trial among adults on MHD. The experimental intervention consisted of typical multicomponent yoga, including movement, breathing, and meditation modified for intradialysis practice. We achieved our enrollment goal for the study and observed very few adverse events from yoga practice. Despite the high comorbidity and low physical function of patients receiving MHD, the study had high retention and intervention fidelity. Mental health measures from baseline to 12 weeks showed nonsignificant improvements in the yoga group versus the education group. Our results warrant further consideration of multicomponent yoga as a form of physical activity for adults on MHD.

There are limited studies regarding feasibility of conducting a randomized trial of mind-body practices during dialysis. We have previously published preliminary data focused on implementation and refinement of the intervention that allowed us to design and execute this current study. Some studies have looked at tai chi, breathing training, or mindfulness mediation performed during MHD with suggested benefits on mental health and/or physical function.^4^, ^5^, ^6^

Research suggests that conventional physical exercise during MHD is safe and improves quality of life as measured by function and mental health components though there is substantial heterogeneity across studies in effect sizes.^7^ In a meta-analysis, Salhab^7^ reported a mean clinical improvement in mental health of 3.07 (CI 1.29–7.45). Multicomponent yoga is different from conventional exercise. First, the physical intensity of yoga movements is generally lesser than conventional exercise, thereby producing less opportunity for cardiovascular conditioning.^8^ However, the high yoga adherence we observed suggests yoga may have more uptake than conventional exercise among this population, leading to higher overall exercise frequency. Secondly, yoga includes slow breathing exercises, which may have cardiovascular benefits independent of conventional exercise.^9^ Lastly, multicomponent yoga emphasizes relaxation, which provides mental health benefits beyond exercise alone. Patients on MHD have a high burden of anxiety, depression, and chronic pain, and may particularly benefit from yoga. However, in the absence of a direct comparison on health outcomes and safety, it is unclear how IDY compares to conventional exercise.

There were limitations to our study. Participants may have changed behavior outside of dialysis sessions, which was not assessed including practicing yoga, other forms of exercise or mind-body practices, and other health behaviors. Among patients screened for participation, 2 out of 3 were not eligible for participation based on our exclusion criteria. This presumably resulted in a “healthier” patient population than the general patient population receiving MHD. This supports the notion that exercise behavioral interventions are feasible to implement in this otherwise sedentary population with chronic disease. Because we excluded patients deemed high risk, intradialysis yoga may not be applicable to the broader MHD population. We did not collect data regarding detailed adherence to education intervention, which limits direct comparison of patient engagement between groups. A longer intervention than 12 weeks may be necessary to see the impact of the intervention. As a pilot study, the study did not have adequate statistical power to assess changes in primary outcomes of quality of life.

In conclusion, this pilot study demonstrates the feasibility, and importantly supports the safety of conducting a parallel group randomized trial of intradialysis multicomponent yoga as a form of relaxation and exercise in MHD patients. Larger clinical trials with adequate sample size may be performed in the future to examine potential health benefits for quality of life, including symptom management and mental health.

## Disclosure

All authors declared no competing interests.

The content is solely the responsibility of the authors and does not necessarily represent the official views of the National Institutes of Health.

## Supplementary Material

**Supplementary Methods.**

**Table S1.** Twelve-week intradialysis yoga intervention.

**Table S2.** Participant sociodemographics.

**CONsolidated Standards of Reporting Trial Checklist**
