# Supplementary material for: A Pilot Randomized Trial of Intradialysis Yoga for Patients With End-Stage Kidney Disease
Source: Kidney Int Rep. 2022 Nov 19;8(2):357–9. doi: 10.1016/j.ekir.2022.11.007 (PMC9939353; doi:10.1016/j.ekir.2022.11.007)
Supplement: Supplementary File (PDF) [file mmc1.pdf]

METHODS

Study Design

This was a 12-week prospective, randomized, single-blinded, controlled pilot trial performed in MHD patients comparing IDY with health education versus health education alone. Prior to randomization, we performed baseline testing and stratified patients based on age (≥ or < 50 years), BMI (≥ or < 25 m^2/kg), dialysis schedule (Monday, Wednesday, and Friday or Tuesday, Thursday, and Saturday), and dialysis shift (morning, afternoon, and evening) to minimize differences between groups for these variables. Participants were randomized prior to recruitment to either intra-dialysis yoga or the educational program. Treatment assignments were generated by a permuted blocks method randomly varying block size to 2 and 4. Assignments were sealed in numbered, opaque envelopes and opened by research staff before initiating recruitment. The study was approved by the institutional review board, and signed informed consents were obtained from all patients.

Participants

Patients were recruited from Vanderbilt University Medical Center (VUMC) outpatient dialysis clinics in Nashville Tennessee. Potential participants were identified through screening of the dialysis clinic appointment lists and through direct referrals from medical providers. Inclusion criteria were MHD for ≥ 3 months with last Kt/V ≥ 1.2, expected to remain on MHD for at least 6 months, expected to remain in present MHD shift for at least 4 months, and aged ≥ 18 years. Exclusion criteria included acute or chronic medical conditions that would make intra-dialysis yoga potentially hazardous; unstable cardiac disease (angina, life threatening arrhythmias); chronic lung disease that prevents gentle exercise or deep breathing exercises; active cerebrovascular disease; major depression; chronic symptoms of nausea, vomiting, or diarrhea; cognitive impairment (mini-mental exam ≤ 24); current participation in an exercise or mind-body program or class; and subjects that had participated in an earlier safety pilot study of IDY. Patients were offered $200 at the completion of the study.

Randomization

All randomization occurred after baseline testing and stratified based on age (≥ or <50 years) and BMI ((≥ or <25 m^2/kg) to minimize differences between groups for these variables. Treatment assignments will be generated by a permuted blocks method randomly varying block size to 2 and 4. Assignments will be sealed in numbered, opaque envelopes. The study statistician performed randomization.

Interventions

Intradialytic Yoga

The intradialytic yoga protocol has previously been described in detail [22] and summary is shown in Table 1. In brief, participants in the IDY protocol were offered yoga instruction and practice three times a week for 12 weeks. Yoga was offered during the first two hours of each dialysis session. The intervention involved slow body movements coordinated with breathing exercises. Patients were guided through movements that were modified for patient comfort and capacity. Effort was made to not interfere with the administration of MHD. None of the exercises involved movement of the vascular access site. The chair was reclined for some movements and upright for others. The exercises were designed to be

gradual and progressive over 12 weeks to ensure patient safety and allow sustainable behavioral and physical changes. Yoga practices initially were 15 minutes and progressed up to 60 minutes in length depending on the ability of the participant. Each participant was provided modified practices if they were unable to perform a particular exercise throughout the study or during a specific session. In addition, patients used meditation and visualization techniques to enhance relaxation. Participants were taught together as a group in specific areas of the dialysis unit, or individually when this was not feasible. Reasons for not being able to move a patient for group instruction included patient preference and clinical grouping of patients for infection control and contact precautions. All yoga sessions were taught by certified yoga instructors that had completed yoga teacher training programs and received an additional 200 hours or more training in Viniyoga style. The yoga instructors had previously undergone training to deliver IDY, participated in a feasibility study, and demonstrated high fidelity in IDY instruction.

Educational Program

The control group was given an educational course called Kidney School that consisted of 12 modules written at the 6th grade reading level[23]. This educational program has been described in more detail previously [22]. Modules were divided across 12 weeks to match the time of the yoga intervention for each session. Participants had the opportunity to work on these self-directed, paper modules during the first 2 hours of dialysis. The modules contained interactive questions to allow participants to evaluate and personalize their learning experience with entries that were incorporated into personal action plans. Usual care of patients continued as per routine management and protocol of the dialysis centers.

Masking

Consents to participate in the study described interventions as healthy lifestyle changes including exercise, diet, and relaxation techniques without stating or specifying yoga or mind-body practices. Data collection in the form of questionnaires was performed by a research assistant blinded to treatment assignment. The research assistant also administered the 6-minute walk test and extracted additional data from the medical chart regarding medications, dialysis adequacy, labs, pre- and MHD vital signs. Analyses were performed by statisticians blinded to treatment assignments.

Outcome Assessment

The primary outcome of this pilot study was the self-reported physical functioning at 12 weeks. This was determined by (KDQOL), a patient questionnaire containing generic and specific disease cores that measure physical and mental health to determine health-related quality of life [24]. Secondary outcomes included self-reported mental health, physical performance, and self-efficacy. Mental health was assessed using the KDQOL mental health score [24]. Physical performance was assessed using the 6-minute walk test, which measures the distance the subject is able to walk self-paced for six minutes and has been used previously to quantify physical functioning in this patient population [25]. Self-efficacy was assessed using a modified version of the Perceived Medical Condition Self-Management Scale (PDMS), an 8-item tool that measures general self-efficacy [26]. Measurements for each outcome occurred at baseline and at the end of the 12-week study period, as well as at 6 weeks for KDQOL physical function and mental health. Measurements were collected by trained research assistants.

Safety and adverse events were monitored both during IDY sessions and between MHD treatments through documentation of routine vital signs, vascular access dysfunction, hypotensive or hypertensive

episodes, muscle cramps, dizziness, musculoskeletal injuries, cardiovascular events, hospitalizations, or deaths.

Statistical Analyses

All analyses were performed as intention-to-treat. Population characteristics were described overall and by intervention group using median [inter-quartile range] for continuous variables and frequency (%) for categorical variables. Ordinary least squares regression was used to examine the relationship between four outcomes (KDQOL physical function, KDQOL mental function, physical performance, PDMS) and intervention group (yoga, education). Models were adjusted for baseline score, gender and diabetes status. Baseline score was modeled using restricted cubic splines with 3 knots to allow for potential non-linearity. KDQOL physical and mental function outcomes were defined as the sum of available 6-week and 12-week scores. The implications of using available follow-up measurements are discussed in the limitation subsection. Physical function was defined using the 12-week score (distance) of a 6-minute walk test and PDSMS self-efficacy was overall score at 12 weeks. Test of covariate or treatment effects were calculated with F statistics. Data were analyzed using R version 3.6.3. As a pilot study, a sample size was based on assessing feasibility not KDQOL measurements.

Table S1: **12-week Intra-dialysis Yoga Intervention**

**Week**		**1**	**2**	**3**	**4/5**	**6/7**	**8**	**9/ 10**	**11/ 12**
**Postures**									
**Reclined chair**									
• Hip flexion	With knees bent, one or two knees brought towards chest as hip flexes	✓	✓	✓	✓	✓	✓	✓	✓
• Hip twist	With knees bent, both knees move side to side while upper torso stays flat	✓	✓	✓	✓	✓	✓	✓	✓
• Anterior arm extension	One arm raised overhead from the front	✓	✓	✓	✓	✓	✓	✓	✓
• Rest	No movement	✓	✓	✓	✓	✓	✓	✓	✓
• Hip abduction	With knees bent, one knee is opened towards side (half-butterfly)		✓	✓	✓	✓	✓	✓	✓
• Knee extension	With knees bent, one knee is extended upwards towards a straight leg			✓	✓	✓	✓	✓	✓
**Upright chair**									
• Knee extension	With knees bent, one knee is extended straight			✓	✓	✓	✓	✓	
• Knee extension and ankle-flexion	After knee is extended, ankle is extended and then flexed								✓
• Arm extension	One arm raised overhead from the the front					✓	✓	✓	✓
• Ankle flexion	Ankle flexion and extended					✓	✓	✓	✓
• Forward bend	Bending torso forward over knees					✓	✓	✓	✓
• Chest expansion	Start with flexed spine (“slouching”) and gradually extended spine into arch						✓	✓	✓

• Upper-thoracic twist	Rotate upper thoracic spine while rotating head in same direction							✓	✓
**Breathing**									
• Free observed breath		✓	✓						
• Controlled breathing: Inspiration<expiration				✓	✓	✓	✓	✓	✓
• Cooling breath: Inspiration<expiration					✓	✓	✓	✓	✓
**Meditation**									
• Visualize pleasant moving water		✓							
• Visualize pleasant moving water over body			✓						
• Visualize moving water with hand from abdomen to chest, and from chest away from body				✓	✓	✓	✓	✓	✓
• Visualize water moving with hand into body, up and down body, and then from chest away					✓	✓	✓	✓	✓

Table S2: Participant Sociodemographics[1]

	**Yoga** (n = 34)	**Education** (n = 33)
Age	58 [50, 64]	58 [51, 64]
Gender		
Female	44% (15)	64% (21)
Race		
African American	94% (32)	82% (27)
White	3% (1)	3% (1)
Other (e.g. Asian)	3% (1)	15% (5)
Education		
Some high school/high school	41% (14)	58% (19)
Some college/2 year college	29% (10)	33% (11)
4 year college or more	29% (10)	6% (2)
Smoking		
Current	21% (7)	27% (9)
Previous	29% (10)	15% (5)
Never	50% (17)	58% (19)
Years on HD	4.5 [3.0, 6.0]	2.0 [1.0, 6.0]
Cause of ESRD		
Hypertension	50% (17)	55% (18)
Diabetes	38% (13)	15% (5)
Other	12% (4)	30% (10)
History of transplant		
Yes	9% (3)	18% (6)
Access		
Catheter	21% (7)	15% (5)
Arteriovenous fistula or graft	79% (27)	85% (28)

CONSORT 2010 checklist of information to include when reporting a randomised trial*

Section/Topic	Item No	Checklist item	Reported on page No
**Title and abstract**			
	1a	Identification as a randomised trial in the title	1
	1b	Structured summary of trial design, methods, results, and conclusions (for specific guidance see CONSORT for abstracts)	2
**Introduction**			
Background and objectives	2a	Scientific background and explanation of rationale	1-2
	2b	Specific objectives or hypotheses	2
**Methods**			
Trial design	3a	Description of trial design (such as parallel, factorial) including allocation ratio	S1
	3b	Important changes to methods after trial commencement (such as eligibility criteria), with reasons	n/a
Participants	4a	Eligibility criteria for participants	S1
	4b	Settings and locations where the data were collected	S1
Interventions	5	The interventions for each group with sufficient details to allow replication, including how and when they were actually administered	S1-S2
Outcomes	6a	Completely defined pre-specified primary and secondary outcome measures, including how and when they were assessed	S2
	6b	Any changes to trial outcomes after the trial commenced, with reasons	n/a
Sample size	7a	How sample size was determined	S2
	7b	When applicable, explanation of any interim analyses and stopping guidelines	n/a
Randomisation:			S1

Sequence generation	8a	Method used to generate the random allocation sequence	
	8b	Type of randomisation; details of any restriction (such as blocking and block size)	S1
Allocation concealment mechanism	9	Mechanism used to implement the random allocation sequence (such as sequentially numbered containers), describing any steps taken to conceal the sequence until interventions were assigned	S1
Implementation	10	Who generated the random allocation sequence, who enrolled participants, and who assigned participants to interventions	S1
Blinding	11a	If done, who was blinded after assignment to interventions (for example, participants, care providers, those assessing outcomes) and how	S2
	11b	If relevant, description of the similarity of interventions	
Statistical methods	12a	Statistical methods used to compare groups for primary and secondary outcomes	S2
	12b	Methods for additional analyses, such as subgroup analyses and adjusted analyses	S2
**Results**			
Participant flow (a diagram is strongly recommended)	13a	For each group, the numbers of participants who were randomly assigned, received intended treatment, and were analysed for the primary outcome	Fig 1
	13b	For each group, losses and exclusions after randomisation, together with reasons	Fig 1
Recruitment	14a	Dates defining the periods of recruitment and follow-up	2
	14b	Why the trial ended or was stopped	Completed,
Baseline data	15	A table showing baseline demographic and clinical characteristics for each group	Table S2
Numbers analysed	16	For each group, number of participants (denominator) included in each analysis and whether the analysis was by original assigned groups	2

Outcomes and estimation	17a	For each primary and secondary outcome, results for each group, and the estimated effect size and its precision (such as 95% confidence interval)	2
	17b	For binary outcomes, presentation of both absolute and relative effect sizes is recommended	n/a
Ancillary analyses	18	Results of any other analyses performed, including subgroup analyses and adjusted analyses, distinguishing pre-specified from exploratory	2
Harms	19	All important harms or unintended effects in each group (for specific guidance see CONSORT for harms)	2
**Discussion**			
Limitations	20	Trial limitations, addressing sources of potential bias, imprecision, and, if relevant, multiplicity of analyses	3
Generalisability	21	Generalisability (external validity, applicability) of the trial findings	3
Interpretation	22	Interpretation consistent with results, balancing benefits and harms, and considering other relevant evidence	3
**Other information**			
Registration	23	Registration number and name of trial registry	1
Protocol	24	Where the full trial protocol can be accessed, if available	n/a
Funding	25	Sources of funding and other support (such as supply of drugs), role of funders	6
